# DFT Prediction of Factors Affecting the Structural Characteristics, the Transition Temperature and the Electronic Density of Some New Conjugated Polymers

**DOI:** 10.3390/polym12061207

**Published:** 2020-05-26

**Authors:** Quoc-Trung Vu, Thi-Thuy-Duong Tran, Thuy-Chinh Nguyen, Thien Vuong Nguyen, Hien Nguyen, Pham Van Vinh, Dung Nguyen-Trong, Nguyen Dinh Duc, Phuong Nguyen-Tri

**Affiliations:** 1 Faculty of Chemistry, Hanoi National University of Education, 136 Xuan Thuy, Cau Giay, Hanoi 180000, Vietnam; tttthuyduong93@gmail.com (T.-T.-D.T.); hiennguyendhsphn@gmail.com (H.N.); 2 Institute for Tropical Technology, Vietnam Academy of Science and Technology, 18, Hoang Quoc Viet, Cau Giay, Hanoi 122300, Vietnam; thuychinhhn@gmail.com (T.-C.N.); vuongvast@gmail.com (T.V.N.); 3 Faculty of Physics, Hanoi National University of Education, 136 Xuan Thuy, Cau Giay, Hanoi 100000, Vietnam; vinhpv@hnue.edu.vn (P.V.V.); dungntsphn@hnue.edu.vn (D.N.-T.); 4 Faculty of Environmental and Food Engineering, Nguyen Tat Thanh University, 300A Nguyen Tat Thanh, District 4, Ho Chi Minh City 755414, Vietnam; nguyensyduc@gmail.com; 5 Department of Environmental Energy Engineering, Kyonggi University, Suwon 16227, Korea; 6 Institute of Research and Development, Duy Tan University, Da Nang 550000, Vietnam; 7 Department of Chemistry, Biochemistry and Physics, University of Quebec in Trois-Rivieres (UQTR), Trois-Rivieres, QC G8Z 4M3, Canada

**Keywords:** conjugated polymers, density functional theory, bond length, transition temperatures

## Abstract

Conjugated polymers are promising materials for various cutting-edge technologies, especially for organic conducting materials and in the energy field. In this work, we have synthesized a new conjugated polymer and investigated the effect of distance between bond layers, side-chain functional groups (H, Br, OH, OCH_3_ and OC_2_H_5_) on structural characteristics, phase transition temperature (T), and electrical structure of C_13_H_8_OS using Density Functional Theory (DFT). The structural characteristics were determined by the shape, network constant (a, b and c), bond length (C–C, C–H, C–O, C–S, C–Br and O–H), phase transition temperatures, and the total energy (E_tot_) on a base cell. Our finding shows that the increase of layer thickness (h) of C_13_H_8_OS–H has a negligible effect on the transition temperature, while the energy bandgap (E_g_) increases from 1.646 eV to 1.675 eV. The calculation of bond length with different side chain groups was carried out for which C_13_H_8_OS–H has C–H = 1.09 Å; C_13_H_8_OS–Br has C–Br = 1.93 Å; C_13_H_8_OS–OH has C–O = 1.36 Å, O–H = 0.78 Å; C_13_H_8_OS–OCH_3_ has C–O = 1.44 Å, O–H =1.10 Å; C_13_H_8_OS–OC_2_H_5_ has C–O = 1.45 Å, C–C = 1.51Å, C–H = 1.10 Å. The transition temperature (T) for C_13_H_8_OS–H was 500 K < T < 562 K; C_13_H_8_OS–Br was 442 K < T < 512 K; C_13_H_8_OS–OH was 487 K < T < 543 K; C_13_H_8_OS–OCH_3_ was 492 K < T < 558 K; and C_13_H_8_OS–OC_2_H_5_ was 492 K < T < 572 K. The energy bandgap (E_g_) of Br is of E_g_ = 1.621 eV, the doping of side chain groups H, OH, OCH_3,_ and OC_2_H_5_, leads to an increase of E_g_ from 1.621 eV to 1.646, 1.697, 1.920, and 2.04 eV, respectively.

## 1. Introduction

In recent years, conjugated polymers have been being widely studied and used in science and technology as well as in semiconductor devices [1,2], sensors [3,4,5,6,7,8,9], batteries [10,11], super- capacitors [10,12], electromagnetic shielding materials [13,14], and corrosion-resistant materials [15,16,17,18,19,20]. The properties of these materials may be greatly affected by doping/introduction of various substituents groups due to a modification of the electronic density of the molecules. Polypyrrole (PPr) is one of the most popular electrically-conducting polymers [21]. Some experimental studies on the electrical density showed that a pyrrole cation contains four pyrrole units. Bredas et al. using the Hartree Fock method and the STO-3G-based kit suggested that pyrrole did not have Na dopant [22]. With the theoretical method, the use of quantum calculations by the ab initio methods at a simple level cannot accurately describe the electronic structure of polypyrrole. 

Recently, only a few studies have used the Density Functional Theory (DFT) method to study the electronic structure [23,24,25,26,27]. The electronic structure results show that the influence of the forbidden bandwidth on the impurity concentration and E_g_ can be adjusted by doping with different atoms [28,29,30,31]. Thereby, if the polymer is doped with an appropriate concentration, it can switch from being a semiconductor to a metal or back to an insulating material [32,33,34,35]. This has attracted particular attention from researchers to determine the transition between conductors and insulators such as transistors, light-emitting diodes, and solar cells [36]. For this purpose, it is necessary to control the bandgap doping. Some recent studies have shown promising results based on calculations through the original principles [37,38,39,40,41,42,43,44]. 

Rittmeyer et.al successfully used DFT to study the derived C_13_H_8_OS in which an H atom was replaced by functional groups, viz. CH_3_, NH_2_, NO_2_, and Cl. The results showed a significant influence of the substitutional elements on the bandgap. The absorption spectrum showed that the bandgap and optical properties are closely related to the shape of the thiophene molecule [45,46]. Most recently, our research group empirically studied C_13_H_8_OS–X monomers (X = H, Br, OH, OCH_3_ and OC_2_H_5_) that were synthesized from thiophene-3-carbaldehyde [47]. Their structures were confirmed by FTIR, ^1^H-NMR, and ^13^C-NMR spectroscopy. The crystal and molecular structures of C_13_H_8_OS–H, C_13_H_8_OS–Br, C_13_H_8_OS–OH, C_13_H_8_OS–OCH_3_, and C_13_H_8_OS–OC_2_H_5_ were characterized by X-ray diffraction. We showed that the chemical polymerization of monomers C_13_H_8_OS–FeCl_3_ in chloroform had been performed, as recently reported [48,49,50,51]. The obtained results show that the bonding lengths between atoms 1 and 2 are: C–C with Br (1.33 Å), OH (1.33 Å), OCH_3_ (1.33 Å), and OC_2_H_5_ (1.32 Å); as well as C–O with Br (1.22 Å), OH (1.23 Å), OCH_3_ (1.23 Å), and OC_2_H_5_ (1.22 Å) [47]. However, the effect of doping structure on the structural shape, the transition temperature and the electronic structure of the monomer C_13_H_8_OS–X (X are: H, Br, OH, OCH_3_, OC_2_H_5_) is still unknown. The main goal of this work is to answer to this fundamental and important question by/using DFT method, which have been successfully used for various materials.

## 2. Method of Calculation

**Scheme 1:**
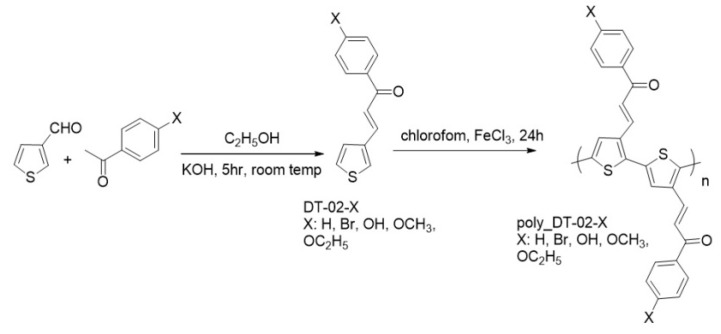
The synthetic procedure of poly(C_13_H_8_OS–X), X is H, Br, OH, CH_3_, C_2_H_5_.

Scheme 1 presents the synthetic procedure of the poly (C_13_H_8_OS–X) where X are H, Br, OH, OCH_3_ and OC_2_H_5_. To study the structural characteristics, transition temperatures and electronic structure of poly[3-(3-phenyl prop-1-ene-3-one-1-yl)thiophene], DFT [52,53,54,55] with the DMol3 module [54] of the copyrighted Material Studio software, a commercial software package installed at the Center for a Computational Science of the Hanoi University of Education (Hanoi, Vietnam) was used. This is a modeling and simulation with the GGA package [56] for which the parameters of the PW91 exchange-correlation function [57,58] and the K-point grid sampling of the diagram Monkhorst-Pack [59] were set into a tridimensional cell unit with a defined dimensions a, b, and c as follows: poly(C_13_H_8_OS–H) (a = 20 Å, b = 13 Å, c = 6 Å), C_13_H_8_OS–Br (a = 26 Å, b = 13 Å, c = 6 Å), poly(C_13_H_8_OS–OH) (a = 26 Å, b = 13 Å, c = 6 Å), C_13_H_8_OS–OCH_3_ (a = 29 Å, b = 13 Å, c = 6 Å) and C_13_H_8_OS–OC_2_H_5_ (a = 32 Å, b = 13 Å, c = 6 Å). The electrons interact with each other through the Density Function Semi-core Pseudo-Potential [60] and thus the electrons are considered in a homogeneous state. The energy was set at 1 × 10^−6^ eV, the displacement during the geometry optimization is at level 1 × 10^−5^ Ha/integer, and 5 × 10^−3^ Å. 

The DFT methods [61,62] have been established based on following approaches: Schrodinger model [63,64], Hartree-Fock model [65,66], Thomas-Fermi model [63], Hohenberg theorem [63,67,68] and traditional Kohn-Sham Theory [63,67,69]. To verify the accuracy of results, other methods such as Linear-Muffin-Tin-Orbital (LMTO) [70] method, Korringa-Kohn-Rostocker (KKR) methods [71] and General gradient approximation method (GGA) [72] has been reported.

## 3. Results and Discussion

### 3.1. Effect of Distance between Layers

**Figure 1:**
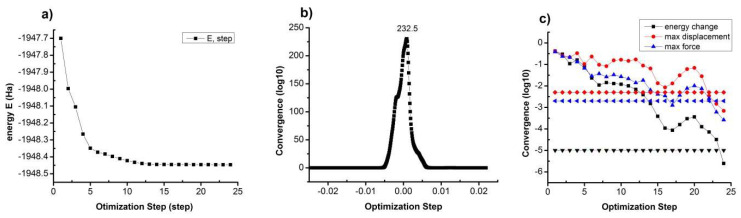
System energy (**a**), electronic density (**b**), electronic state (**c**) of poly(C_13_H_8_OS–H) with a different number of steps.

Initially, all C_13_H_8_OS–X, X = H, Br, OH, CH_3_ and C_2_H_5_ samples were run optimally so that all samples were returned to equilibrium. The results obtained for poly(C_13_H_8_OS–H) are shown in Figure 1.

**Figure 2:**
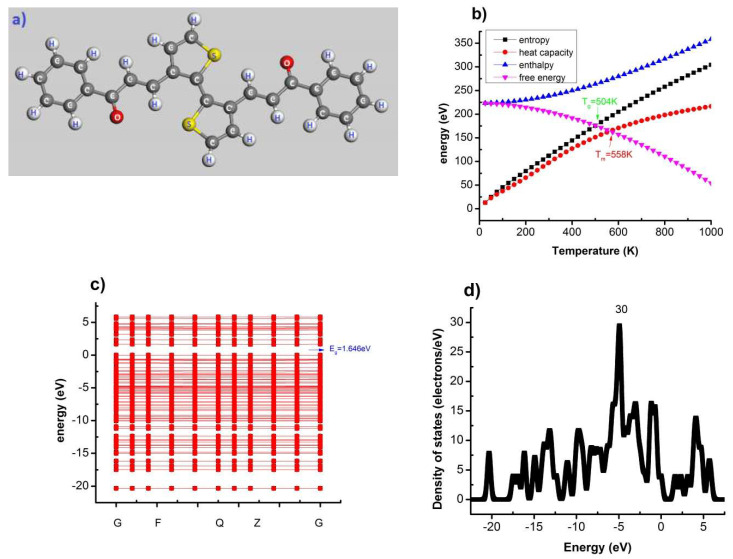
The quantities characteristic of the structure, and electronic structure of poly(C_13_H_8_OS–H) materials such as Shape (**a**), phase transition temperature zone (**b**), electronic structure (**c**), and the density of states (**d**).

Figure 1 shows that with C_13_H_8_OS–H, when increasing the number of steps, the total energy of the system (E_tot_) will decrease from E_tot_ = −1947.7 eV to E_tot_ = −1948.4 eV (Figure 1a). There is a change in electronic density. The maximum value at the equilibrium position is of 232.5 eV (Figure 1b) and the electronic state decreases (Figure 1c) with the number of steps. The results show also that if the number of steps increases, the material will change from the initial state to the equilibrium state. The structural and electronic characteristics of materials in equilibrium state as a function of step number is conducted with a thickness of layers (h), h = 6 Å. The obtained results are presented in Figure 2 and summarized in Table 1.

Figure 2 shows that the shape of C_13_H_8_OS–X after stable running NVE has C_13_H_8_OS–H poly- structures and the atoms C, H, S, O are arranged tightly in a stable triclinic 3D structure. At the same time, the distance between atoms with the change of values by cell size: a = 24 Å, b = 13 Å, c = 6 Å, α = β = γ = 90^o^ the distance between atoms with the first round C–C varies from 1.39 Å to 1.41 Å, C–H: 1.09 Å. The interval between round one and round two: C–C: varies from 1.36 Å to 1.50 Å, C–O: 1.25 Å, C–H: 1.09 Å. The obtained results are completely consistent with the structural determination [47] for which C–C = 1.33 Å, C–O =1.23 Å. The second round: C–C = 1.36–1.42 Å, C–S = 1.72 Å, C–H: 1.09 Å, C–H: 1.09 Å. The bond angle of round one: C–C–C: changes from 120.17° to 120.8°, HCC: 120.02°, C–C–C connection interval: 119.21°, C–C–O: 119.7° to 121.08°, C–C–H: 119.26° to 121.16°. The bond angle of round two: C–C–C changes from 120.17° to 120.80° and H–C–C: 120.18°, C–C–C: 93.22°, C–S–C: 114.17°, S–C–C: 108.82°, C–C–H: 122.61°, S–C–H: 119.25° (Figure 2a) and electronic density at different levels of the conduction band (Table 1, Figure 2d). The transition temperature of poly(C_13_H_8_OS–H) materials is found to be 504 K < T < 558 K (Figure 2b). Here T_c_ = 504 K is called the crystalline temperature (the temperature of transferring materials from liquid state to crystalline state), melting point (T_m_ = 558 K) is the phase transition temperature (the temperature of transferring materials from the crystalline state to the liquid state), the width forbidden region is of E_g_ =1.646 eV (Figure 2c), the electronic density in equilibrium has the maximum value of 235 eV (Figure 2d). In particular, the electronic structure features are shown in Figure 2c with a strip structure in the left table and the density of electrons in the right table poly(C_13_H_8_OS–H) is a semiconductor material with a bandgap of E_g_ = 1.646 eV. These results are consistent with the electronic densities of the states on the right of Table 1. 

The results show that at the E = −5 eV the electron density is 29.462%, which shows that in the valence band, the electronic density reaches the maximum value, which proves that poly(C_13_H_8_OS–H) is a semiconductor material. To study the effect of thickness between poly atomic layers, the results are shown in Table 2.

Table 2 shows that, with h = 6 Å, the temperature range of poly(C_13_H_8_OS–H) materials is 504 K < T < 558 K, where T_c_ = 504 K, T_m_ = 558 K, and the energy band gap E_g_ is of 1.646 eV. When the thickness (h) of the atomic layer increases from h = 6 Å to h = 9 Å, 12 Å and 15 Å, the temperature range of poly(C_13_H_8_OS–H) has a negligible change between 504 and 564 K and E_g_ increases from E_g_ = 1.646 eV to E_g_ = 1.675 eV. The bandgap has a constant value when h > 9 Å (Table 2) which indicates that the smaller the atomic distance, the smaller the bandgap. 

**Figure 3:**
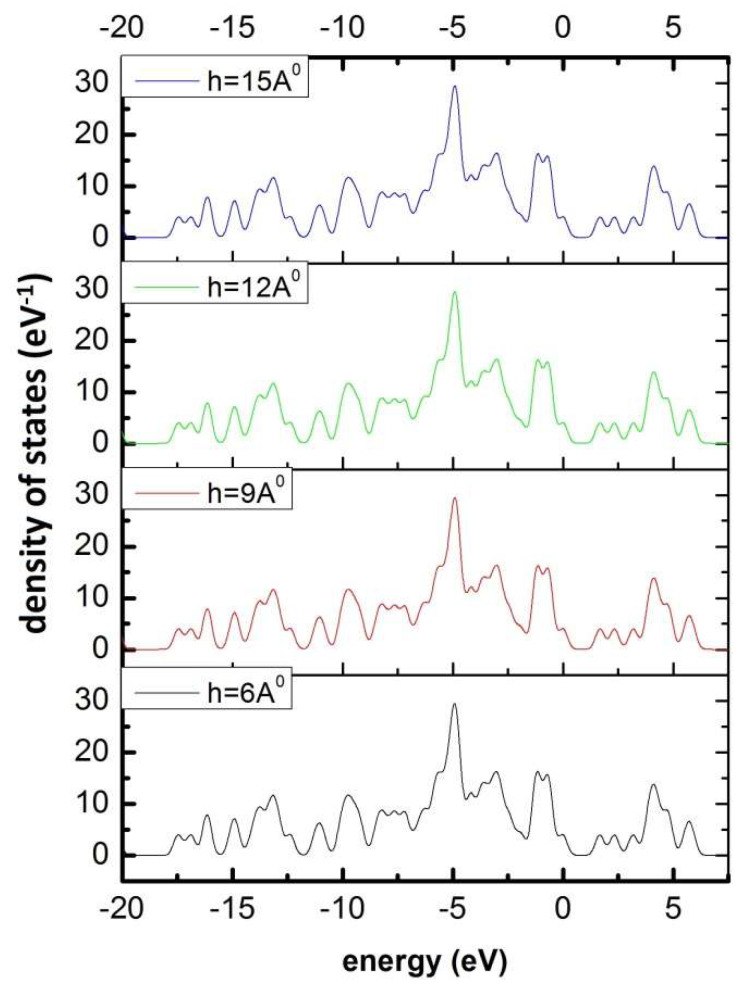
The electronic density in the chemotherapy band.

The increasing distance of the atomic layer leads to E_g_ increases and reaches to a maximum value at h = 9 Å. For more details, the total density of the states of the poly C_13_H_8_OS–H material with increasing thickness of the layers at the density of the states at different energies in the valence band and the conduction band is conducted. The obtained results are shown in Figure 3.

A maximum value of electronic density of poly(C_13_H_8_OS–H) material as a function of atomic layer distance is observed at creating energy band of E = −5 eV, assigned to the electronic density as follows: with h = 6 Å is 29.462%, h = 9 Å, 12 Å, and 15 Å with the electronic density in the valence band has constant value of 29.463%. It turns out that the highest electron density level leads to the least flexible conductivity because the electrons are closely linked to the network node. Increasing the thickness of the atomic layers of poly(C_13_H_8_OS–H) leads to an increase of the density of electrons in the valence band, after that this effect is less pronounced with a slight increase from 4.011% to 4.018%. Electron density not significantly changed in the conduction band at h = 6 Å, in which electron density in the conduction band of poly(C_13_H_8_OS–H) increases at E = 2.5 eV (Figure 3), leading to a decrease of the mobility of electrons in the valence band. This shows an important effect of bond layers, on the structural characteristics, the transition temperature, and electronic properties of poly(C_13_H_8_OS–H).

### 3.2. Effect of Impurities/Heterogeneity

**Figure 4:**
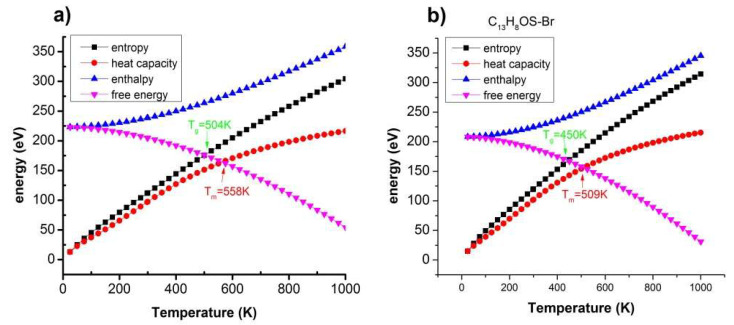

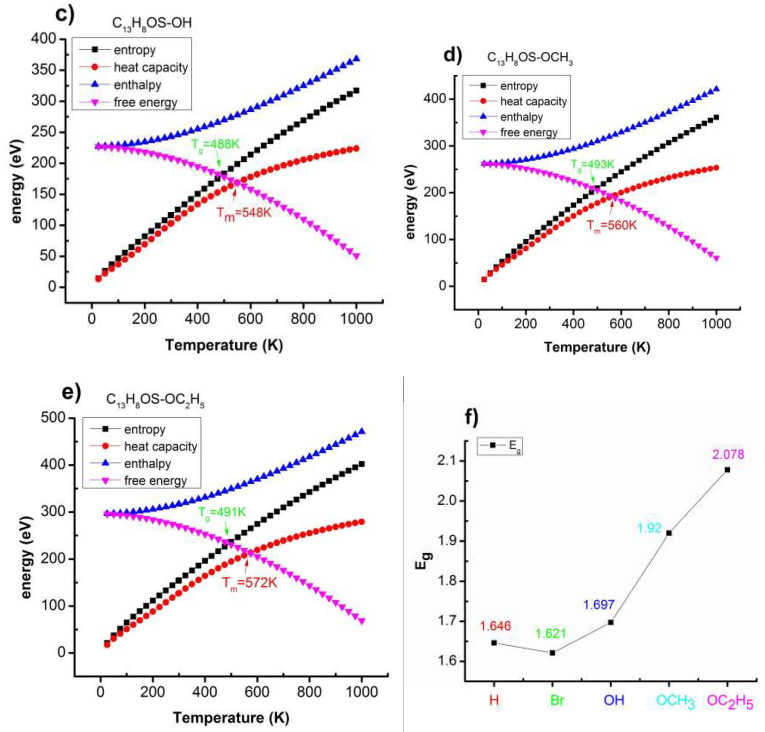
The electronic structures of poly(C_13_H_8_OS–H) (**a**), poly(C_13_H_8_OS–Br) (**b**), poly(C_13_H_8_OS–OH) (**c**), poly(C_13_H_8_OS–OCH_3_) (**d**), and poly(C_13_H_8_OS–OC_2_H_5_) (**e**), E_g_ of poly with different impurities (**f**).

When C_13_H_8_OS–X is doped/modified by different atoms or functional groups: H, Br, OH, OCH_3,_ and OC_2_H_5_, the different energies of the molecule, the structure, electronic structure as a function of temperature were calculated and then plotted in Figure 4.

**Figure 5:**
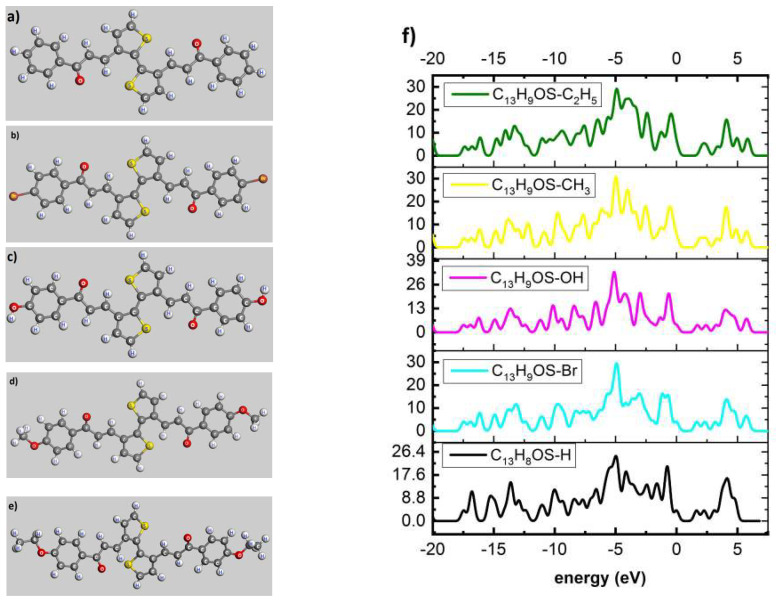
The structural shapes of C_13_H_8_OS–H (**a**), C_13_H_8_OS–Br (**b**), C_13_H_8_OS–OH (**c**), C_13_H_8_OS–OCH_3_ (**d**), C_13_H_8_OS–OC_2_H_5_ (**e**), and the electronic energies structures with poly different (**f**).

The results show that the material shape is poly(C_13_H_8_OS–H) (Figure 4a), with the bandgap of E_g_ = 1.646 eV (Figure 4f). For poly(C_13_H_8_OS–X) material doped/modified with X (X is H, Br, OH, OCH_3_, OC_2_H_5_), the shapes of poly(C_13_H_8_OS–Br), poly(C_13_H_8_OS–OH), poly(C_13_H_8_OS–OCH_3_), and poly(C_13_H_8_OS–OC_2_H_5_) have changed significantly: temperature range of phase transition of poly(C_13_H_8_OS–H) = 504 K < T < 558 K; poly(C_13_H_8_OS–Br) = 450 K < T < 509 K (Figure 4b); poly(C_13_H_8_OS–OH) = 488 K < T < 548 K (Figure 5c); poly(C_13_H_8_OS–OCH_3_) = 493 K < T < 560 K (Figure 4d); poly(C_13_H_8_OS–OC_2_H_5_) = 491 K < T < 572 K (Figure 4e), and the bandgap E_g_ decreases from E_g_ = 1.646 eV to E_g_ = 1.621 eV with poly(C_13_H_8_OS–Br) and E_g_ increases with poly(C_13_H_8_OS–OH) from E_g_ = 1.646 eV to E_g_ = 1.697 eV; E_g_ increased from 1.646 eV to 1.920 eV with poly(C_13_H_8_OS–OCH_3_); E_g_ increased from 1.646 eV to 2.078 eV with poly(C_13_H_8_OS–OCH_3_) (Figure 4f). This shows that the doping poly(C_13_H_8_OS–H) with Br (electrophilic group) leads to a decrease of both T and E_g_ while doping this molecule with nucleophilic groups such as OH, OCH_3,_ and OC_2_H_5_ leads to an increment of T and E_g_. 

The effect of the nature of the substituents/substitution groups on the molecular shape and the electron density of the energy bands are then investigated and the obtained results are shown in Figure 5.

The results show that molecular shape exhibits box-shaped with a precise cell size as follows: C_13_H_8_OS–H: a = 24 Å, b = 133 Å and c = 6 Å; C_13_H_8_OS–Br: a = 26 Å, b = 133 Å, c = 6 Å; poly(C_13_H_8_OS–OH): a = 26 Å, b = 133 Å, c = 6 Å; C_13_H_8_OS–OCH_3_: a = 29 Å, b = 133 Å, c = 6 Å; poly(C_13_H_8_OS–OC_2_H_5_): a = 32 Å, b = 133 Å, c = 6 Å. The bond angles of different poly(C_13_H_8_OS–H) derivatives are also calculated: poly(C_13_H_8_OS–H) with C–C–H = 120.02°; poly(C_13_H_8_OS–Br) with C–C–Br = 119.13°; poly (C_13_H_8_OS–OH) with C–O–H = 109.38°; poly(C_13_H_8_OS–OCH_3_) with C–C–O = 115.62°, O–C–H = 110.56°, H–C–H = 109.66°; poly(C_13_H_8_OS–OC_2_H_5_) with C–O–C = 118.28°, O–C–H = 108.79°, H–C–H = 108.79°, C–C–H = 109.22°. The electronic density of poly(C_13_H_8_OS–H) with energy bands of E = −20, −15, −10, −5, −2.5, 0, 2.5, 5 and 7.5 eV exhibit electric densities equal to 1.728%, 8.574%, 11.993%, 24.814%, 11.434%, 3.945%, 2.022%, 4.182%, and 0%, respectively (Figure 5f). If doping the functional side groups Br, OH, CH_3_, or C_2_H_5_ on C_13_H_8_OS, the electronic density will be greatly changed. For example, for energy in the range of 20 eV, the electron density increases from 1.728% to 2.55%, 4.033%, 5.667%, or 7.325%; for the energy band E = −15 eV, the electron density decreased from 8.57% to 6.02%, 5.91%, 4.83%, or 2.359%; for the E = −10 eV energy range, the electron density decreases from 11.99% to 8.01%. The obtained results show that the distance and the angle between atoms in the aromatic rings did not change significantly for C_13_H_8_OS–H. However, the distance between the atoms of impurities/heterogeneity varies greatly for poly(C_13_H_8_OS–H) doped with C–H (1.09 Å—Figure 5a); poly (C_13_H_8_OS–Br) doped with C–Br = 1.93 Å (Figure 5b); poly(C_13_H_8_OS–OH) doped with C–O = 1.36 Å, O–H = 0.78 Å (Figure 5c); poly(C_13_H_8_OS–OCH_3_) with C–O =1.44 Å, O–H = 1.10 Å (Figure 5d); poly(C_13_H_8_OS–OC_2_H_5_) with C–O = 1.45 Å, C–C is 1.51 Å, C–H, = 1.10 Å (Figure 5e). 

Besides, in the valence region, the electron density accounts for the largest proportion, reaching the extreme value in the energy band E = −5 eV. These results confirmed this is still a semiconductor material and only increasing the conductivity when doping the group Br function leads to a decrease in the E_g_ bandgap, a decrease in conductivity when doping/introducing the OH, CH_3_, or the C_2_H_5_ functional side groups leads to an increase in the E_g_ bandgap (Figure 5f). These results are shown in the first Brillouin region (the level of 0) corresponding to the density of electrons increased from 3.945% to 3.949%, 4.509%, 7.903% and 13.967% and this leads to an increment of the electron mobility of the C_13_H_8_OS substance with Br doping but decreases with H, OH, OCH_3_, and OC_2_H_5_ functional side groups. This confirms the influence of impurities/heterogeneity on the lattice structure and electronic structure of C_13_H_8_OS material. In other words, when increasing the distance between atomic layers, the structural shape, the distance between the atoms, the total energy of the system and the bandgap are almost constant, especially the phase transition temperature and the conductivity of electrons decreases. When doping/fine-tuning with different groups and atoms such as Br, OH, OCH_3_, or OC_2_H_5_, the distance between atoms, the total energy of the system, and the bandgap show a great change (increase the conductivity when Br is doped, reducing the conductivity when it is doped with OH, OCH_3._ or OC_2_H_5_).

## 4. Conclusions

In summary, we report a successful investigation of factors affecting the structural characteristics, the transition temperature, electronic properties of Poly C_13_H_8_OS–X, where X are H, Br, OH, OCH_3_, or OC_2_H_5_ by means of DFT using the GGA-PW91 package. The results showed that the interval between round one and round two: C–C: varies from 1.36 Å to 1.50 Å, C–O: 1.25 Å, C–H: 1.09 Å. The obtained results are completely consistent with the structural determination for which C–C = 1.33 Å, C–O =1.23 Å. The second round: C–C= 1.36–1.42 Å, C–S = 1.72 Å, C–H: 1.09 Å, C–H: 1.09 Å [47]. When increasing the thickness (h) of the atomic layer from h = 6 Å to h = 9, 12 and 15 Å, the transition temperature range of poly(C_13_H_8_OS–H) shows a negligible change value from 504 K < T < 558 K to 504 K < T < 564 K and E_g_ increases from E_g_ = 1.646 eV to E_g_ = 1.675 eV. 

The nature of the substituents (H, Br, OH, CH_3_, C_2_H_5_) in C_13_H_8_OS has a significant effect on the molecular shape and bond length. These values are successfully calculated and reported as follows: C_13_H_8_OS–H with C–H = 1.09 Å; C_13_H_8_OS–Br with C–Br = 1.93 Å; C_13_H_8_OS–OH with C–O = 1.36 Å, O–H = 0.78 Å; C_13_H_8_OS–OCH_3_ with C–O = 1.44 Å, O–H = 1.10 Å; C_13_H_8_OS–OC_2_H_5_ with C–O =1.45 Å, C–C = 1.51 Å, C–H = 1.10 Å. The transition temperatures (T) can also be calculated: C_13_H_8_OS–H: 504 K < T < 558 K; C_13_H_8_OS–Br: 450 K < T < 509 K; C_13_H_8_OS–OH: 488 K < T < 548 K; C_13_H_8_OS–OCH_3_: 493 K < T < 560 K; and C_13_H_8_OS–OC_2_H_5_: 491 K < T < 572 K.

When doping the C_13_H_8_OS with Br the bandgap E_g_ decreases, while the doping/modification with H, OH, OCH_3_, or OC_2_H_5_ leads to an increase of the electrical conductivity in C_13_H_8_OS. Our findings show that all derived C_13_H_8_OS/derivatives still display semiconductor behavior unless/except the case of Br. The precise values of the molecular structure, the chemical bonds, and bond angles provide useful information for future investigations of these new conjugated molecules, especially for potential applications in the energy field and electrical conducting materials based on conjugated polymers.

## Figures and Tables

**Table 1:** The electron density for materials poly(C_13_H_8_OS–H) at different energies in the valence bands.

Poly(C_13_H_8_OS–H)	−20	−15	−10	−5	−2.5	0	2.5	5
Electron density	2.334	6.625	9.423	29.462	8.925	4.011	2.629	4.151

**Table 2:** Electron density for materials poly(C_13_H_8_OS–H) with energies and thickness of layers different in the valence.

d(Å)	6	9	12	15
T(K)	504 < T < 558	504 < T < 564	504 < T < 564	504 < T < 564
E_g_(eV)	1.646	1.675	1.675	1.675
